# Distribution and status of living colonies of *Acropora* spp. in the reef crests of a protected marine area of the Caribbean (Jardines de la Reina National Park, Cuba)

**DOI:** 10.7717/peerj.6470

**Published:** 2019-02-21

**Authors:** Leslie Hernández-Fernández, Roberto González de Zayas, Yunier M. Olivera, Fabián Pina Amargós, Claudia Bustamante López, Lisadys B. Dulce Sotolongo, Fernando Bretos, Tamara Figueredo Martín, Dayli Lladó Cabrera, Francisco Salmón Moret

**Affiliations:** 1 Marine Ecology, Coastal Ecosystem Research Center, Ciego de Avila, Cuba; 2 Department of Tourism and Business, Máximo Gómez Báez University, Ciego de Avila, Cuba; 3 Centro de Estudios Geomáticos, Ambientales y Marinos (GEOMAR), Ciudad de México, México; 4 Environmental Advisors, Avalon-Marlin, Jardines de la Reina, Ciego de Avila, Cuba; 5 Phillip and Patricia Frost Museum of Science, Miami, FL, USA; 6 Coastal Dynamics, Coastal Ecosystem Research Center, Ciego de Avila, Cuba

**Keywords:** Jardines de la Reina National Park, Cuba, *Acropora palmata*, *Acropora cervicornis*, *Acropora prolifera*

## Abstract

The reef crests of the Jardines de la Reina National Park (JRNP) are largely formed by *Acropora palmata*, but colonies of *A. cervicornis* and the hybrid *A. prolifera* are also present. This study shows spatial distribution of colonies, thickets and live fragments of these species in the fore reefs. Snorkeling was used to perform the direct observations. The maximum diameter of 4,399 colonies of *A. palmata* was measured and the health of 3,546 colonies was evaluated. The same was done to 168 colonies of *A. cervicornis* and 104 colonies of *A. prolifera*. The influence of the location and marine currents on a number of living colonies of *A. palmata* was analyzed. For such purpose, reef crests were divided into segments of 500 m. The marine park was divided into two sectors: East and West. The Caballones Channel was used as the reference dividing line. The park was also divided into five reserve zones. We counted 7,276 live colonies of *Acropora* spp. 1.4% was *A. prolifera*, 3.5% *A. cervicornis* and 95.1% *A. palmata*. There were 104 thickets of *A. palmata*, ranging from eight to 12 colonies, and 3,495 fragments; 0.6% was *A. cervicornis* and the rest *A. palmata* (99.4%). In the East sector, 263 colonies (3.8% of the total), six thickets (5.8%) and 32 fragments (1%) of *A. palmate* were recorded. In the same sector, there were 11 fragments (50%) of *A.cervicornis* and two (2%) colonies of *A. prolifera*. Health of *A. palmata* was evaluated as good and not so good in the study area. Health of *A. cervicornis* was critical and health of *A. prolifera* was good in all five reserve zones. There was a significant increase in the number of colonies from east to west (Χ2 = 11.5, gl = 3.0, *p* = 0.009). This corroborates the existence of an important abundance differences between the eastern and the western region of the JRNP. A negative relationship was observed between the number of colonies and the distance from the channel (Χ2 = 65.0, df = 3.0, *p* < 0.001). The influence of the channel, for the live colonies of *A. palmata* is greater within the first 2,000 m. It then decreases until approximately 6,000 m, and no significant increase beyond. The orientation of the reef crests significantly influenced the abundance of the colonies (Χ2 = 15.5, df = 2.9, *p* = 0.001). The results presented here provide a baseline for future research on the status of the populations of *Acropora* spp., considering that there has been a certain recovery of the species *A. palmata* during the last 10–16 years. Given the current status of the populations of *Acropora* spp., conservation actions focusing *A. cervicornis* should be prioritized.

## Introduction

The Jardines de la Reina Archipelago, established as the Jardines de la Reina National Park (JRNP) by the Executive Committee of the Council of Ministers of Cuba in 2010 (6803/2010), has marine and terrestrial ecosystems of high ecological values. Coral reefs are particularly important in the area. In the reef crests, *Acropora palmata* Lamarck, 1816; one of the most representative species of the Caribbean region (Bruckner, 2003), is relatively common. In the reef crests, we also observed colonies of *A. cervicornis* Lamarck, 1816 (Hernández-Fernández, Bustamante-López & Dulce-Sotolongo, 2016) and *A. prolifera* Lamarck, 1816 (L. Hernández-Fernández, C. Bustamante-López & L. B. Dulce-Sotolongo, 2016, personal observation) considered an F1 hybrid of the species *A. palmata* and *A. cervicornis* (Vollmer & Palumbi, 2002). Zlatarski & Martínez-Estalella (1980) described the distribution, variability, taxonomy and associated fauna of *A. palmata* and *A. cervicornis* in Cuba.

The genus *Acropora* is the most diverse reef building coral in the world (Wallace & Rosen, 2006), Florida and the Great Caribbean (Jackson, 1992). This genus significantly contributes to the formation of islands and to coastal protection (Bruckner, 2002). The Atlantic/Caribbean has two species: *A. palmata* and *A. cervicornis*, and also the hybrid *A. prolifera* (National Marine Fisheries Service, 2014). *A. palmata* and *A. cervicornis* were generally the most abundant species in many reefs of the Caribbean. Their high growth rates have allowed these reefs to keep up with changes in sea level. In addition, due to their branching morphologies, they are an important habitat for other reef organisms (Acropora Biological Review Team, 2005), such as fishes, turtles, echinoderms, crustacean and mollusks (Bruckner, 2002). They also provide amazing scenic values for recreational diving.

Both, *A. palmata* and *A. cervicornis,* experienced abrupt declines in their populations in the early 1980s, substantially reducing coral cover and at the same time, their dead skeletons provided substrate for algal growth. Causes of mortality include hurricanes that have affected local populations *Acropora* spp. over the past 20–25 years; also the white-band disease, a more significant cause of mortality over large areas of the Caribbean region (Aronson & Precht, 2001). Decline due to disease has been documented by other studies (Patterson et al., 2002; Muller et al., 2008; Fogarty, 2012, Muller, Rogers & van Woesik, 2014). Such decline has also been attributed to temperature changes that have induced bleaching, physical damage caused by other extreme weather events (Acropora Biological Review Team, 2005), excessive nutrients, overfishing or a combination of these global and local threats (Jackson et al., 2014). *A. palmata* and *A. cervicornis* appear on the "IUCN Red List" as critically endangered (Aronson et al., 2008). In Cuba, *A. palmata* also suffered a massive mortality between the years 1987 and 1992 (Claro, 2007). The large-scale mortality of *A. palmata* affects reef biodiversity, as well as fisheries productivity (Álvarez-Filip et al., 2009).

While some studies have shown recovery of *A. palmata* (Rogers et al., 2002; Zubillaga, Bastidas & Croquer, 2005; Schelten et al., 2006; Zubillaga et al., 2008; Muller, Rogers & van Woesik, 2014; Larson et al., 2014), others have shown little or no recovery (Rodríguez-Martínez et al., 2014; Croquer et al., 2016; Miller, Kerr & Williams, 2016). Alcolado et al. (2003), in a study conducted on the reefs of Cuba, observed high mortality of *A. palmata* in most of the sites along the northern and southern coasts, presumably caused by diseases such as white band, bleaching and white pox.

A thorough study showing the spatial distribution and status of the genus has not been carried out elsewhere in the JRNP, in spite of its importance, threats and current condition. The only reference was the work of Hernández-Fernández & Bustamante-López (2017) on the status of *A. palmata* in four reef crests in the central region of the park. This study describes the distribution and status of live colonies of *Acropora* spp. in the fore reefs of the JRNP.

## Materials and methods

### Study area

**Figure 1:**
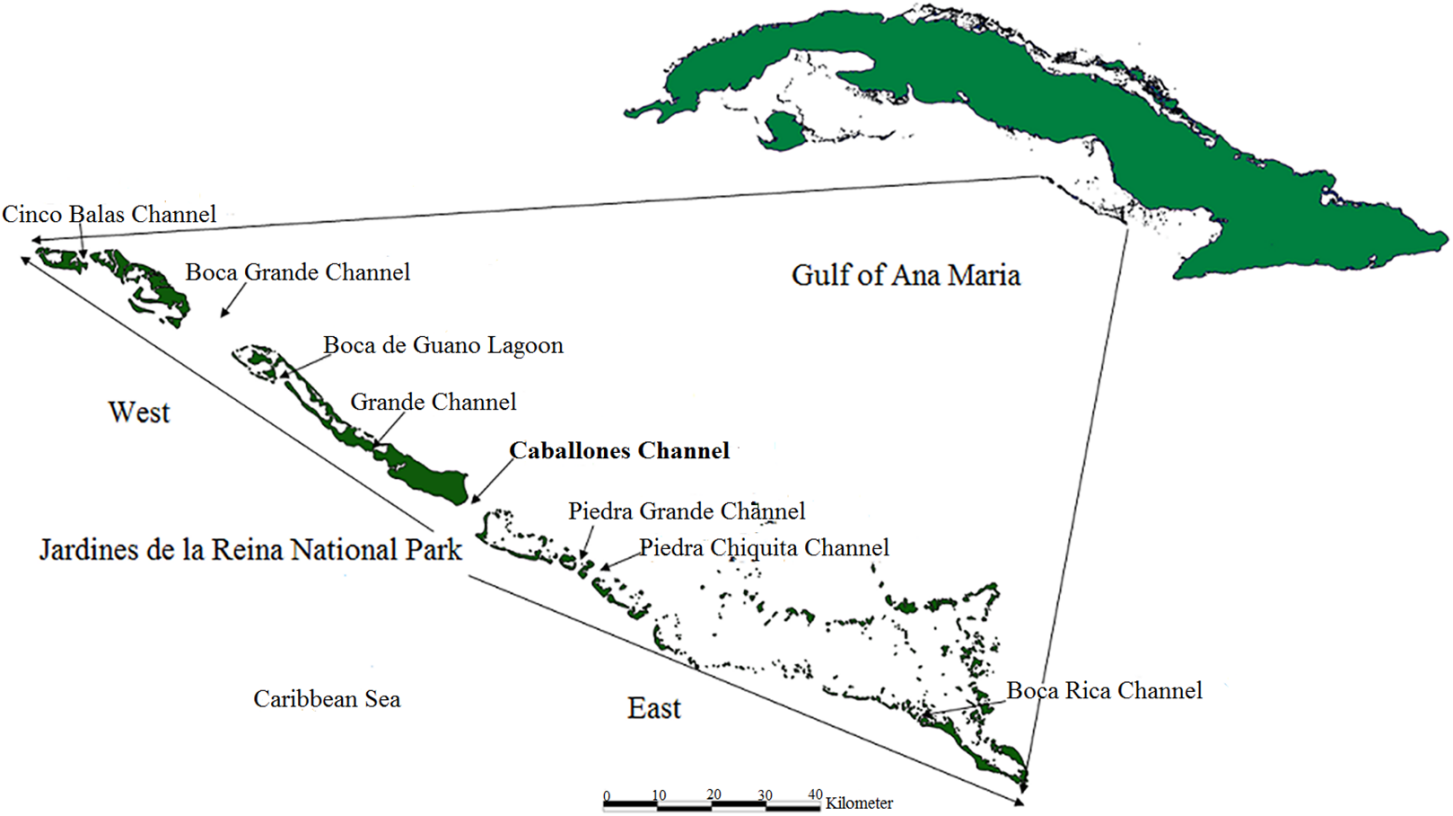
Location of study area; Jardines de la Reina National Park, Cuba.

The distribution and health of colonies, thickets and live fragments of *A. palmata*, *A. cervicornis* and *A. prolifera* were studied in the fore reef zone of the reef crests of the JRNP, which stretches off the southern coast of the provinces of Sancti Spiritus, Ciego de Ávila and Camagüey (Fig. 1).

### Monitoring

The study was conducted in 2017, during the months of August and September. The methodology of Miller, Kerr & Williams (2016), used to determine the abundance and status of *Acropora* spp. populations in the Florida reefs, was also used in this study.

To determine the distribution of colonies, thickets and live fragments of *A. palmata, A. cervicornis* and *A. prolifera*, a direct observation census (snorkeling) was conducted and documented using GPS. Two work teams of six divers were divided into three pairs. Each pair covered an area of up to 500 linear meters in the fore reef zone. The routes, similar to those of Miller, Kerr & Williams (2016), were carried out in zigzags, perpendicular to the reef crest, covering the entire area where colonies, thickets or live fragment of *Acropora* spp. could be found.

The distances covered by each pair were marked with buoys found in most reef crests of the park. The GPSs were wrapped in nylon to prevent water damage and held in ring buoys. Five couples used GARMIN GPS (GPSMAP 78) and one of the couples used GARMIN (Etrex 20). To define a living colony, we considered what Williams, Miller & Kramer (2006) proposed in the monitoring protocol for *Acropora* spp. established for the Caribbean area. "Thickets" were defined when it was not feasible to demarcate individual colonies. At least three points were taken into account to determine the size of the thickets. For fragments, pieces of the colonies were selected, namely broken branches of *Acropora* spp. on the substrate, lacking a defined base (Martínez & Rodríguez-Quintal, 2012).

One member of each pair took the coordinates and the other described the health of the colony, thickets and fragment. This exercise was previously tested. The coordinates taken with the GPS and information gathered were entered into a database upon a daily basis. Spatial distribution was obtained with the program QGIS 2.18. Taking the Caballones Channel as reference, the study area was divided into two sectors (East and West) (Fig. 1).

**Figure 2:**
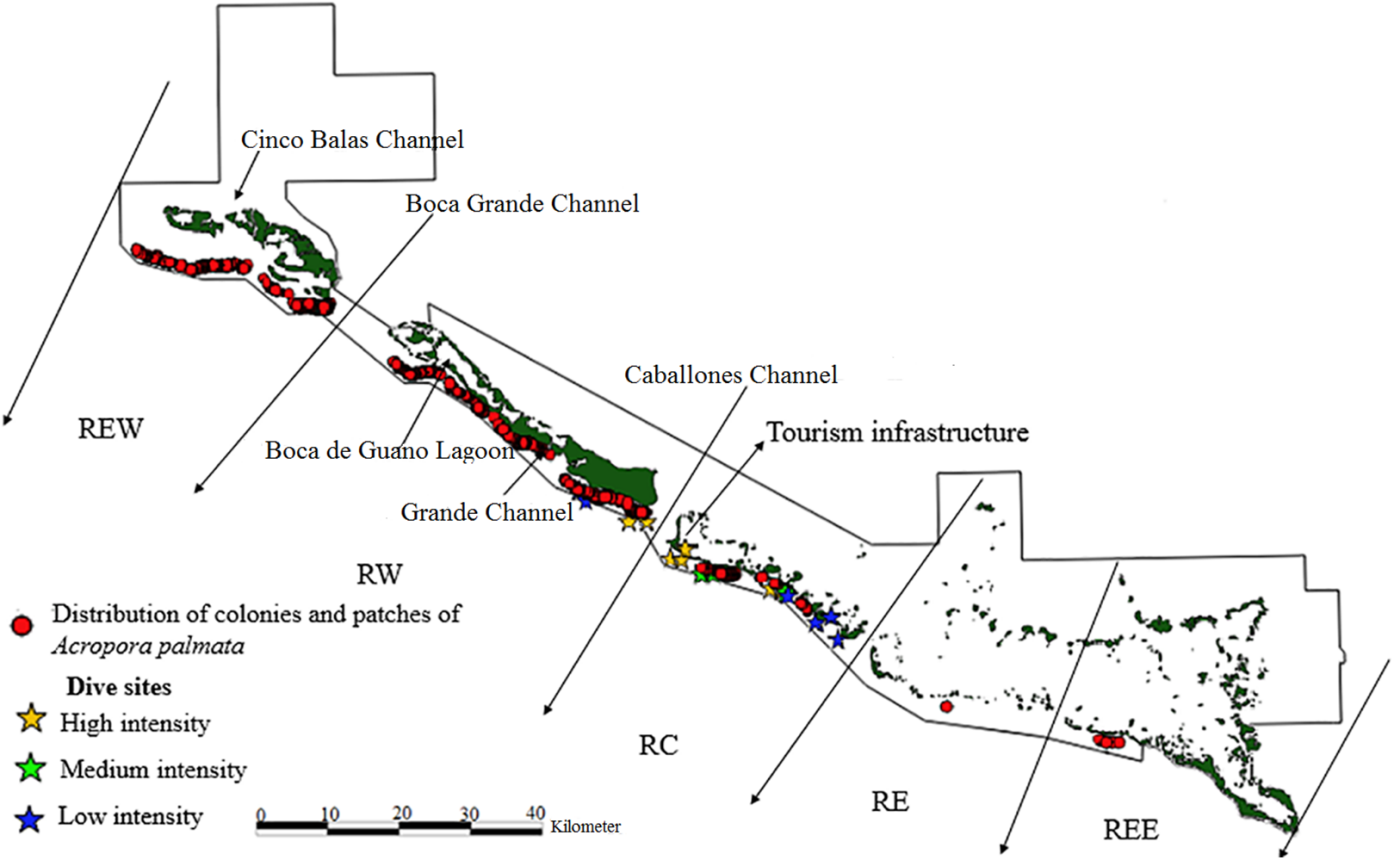
Distribution of live colonies and patches of *Acropora palmata* in Jardines de la Reina National Park, Cuba.

**Figure 3:**
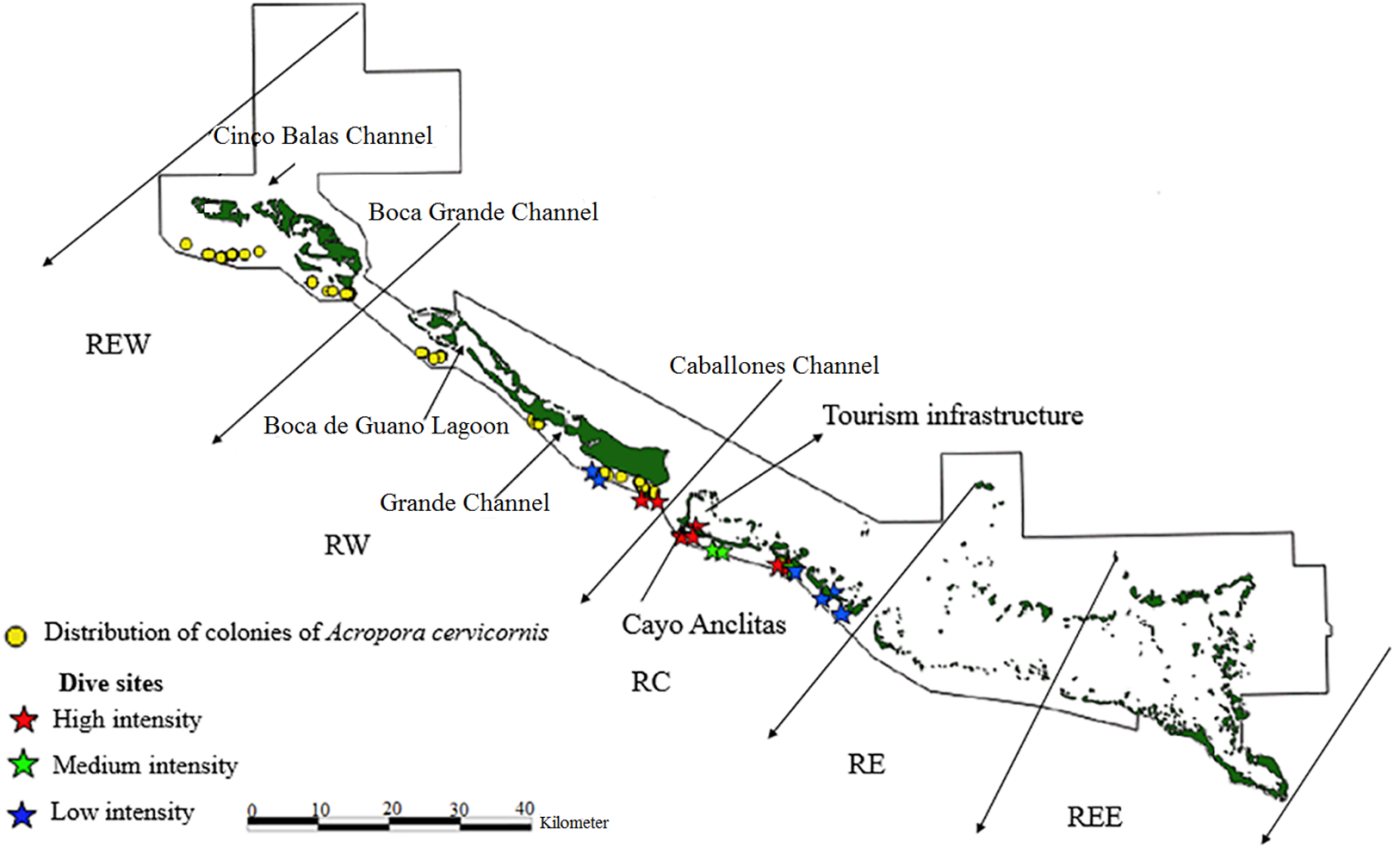
Distribution of live colonies of *Acropora cervicornis* in Jardines de la Reina National Park, Cuba.

**Figure 4:**
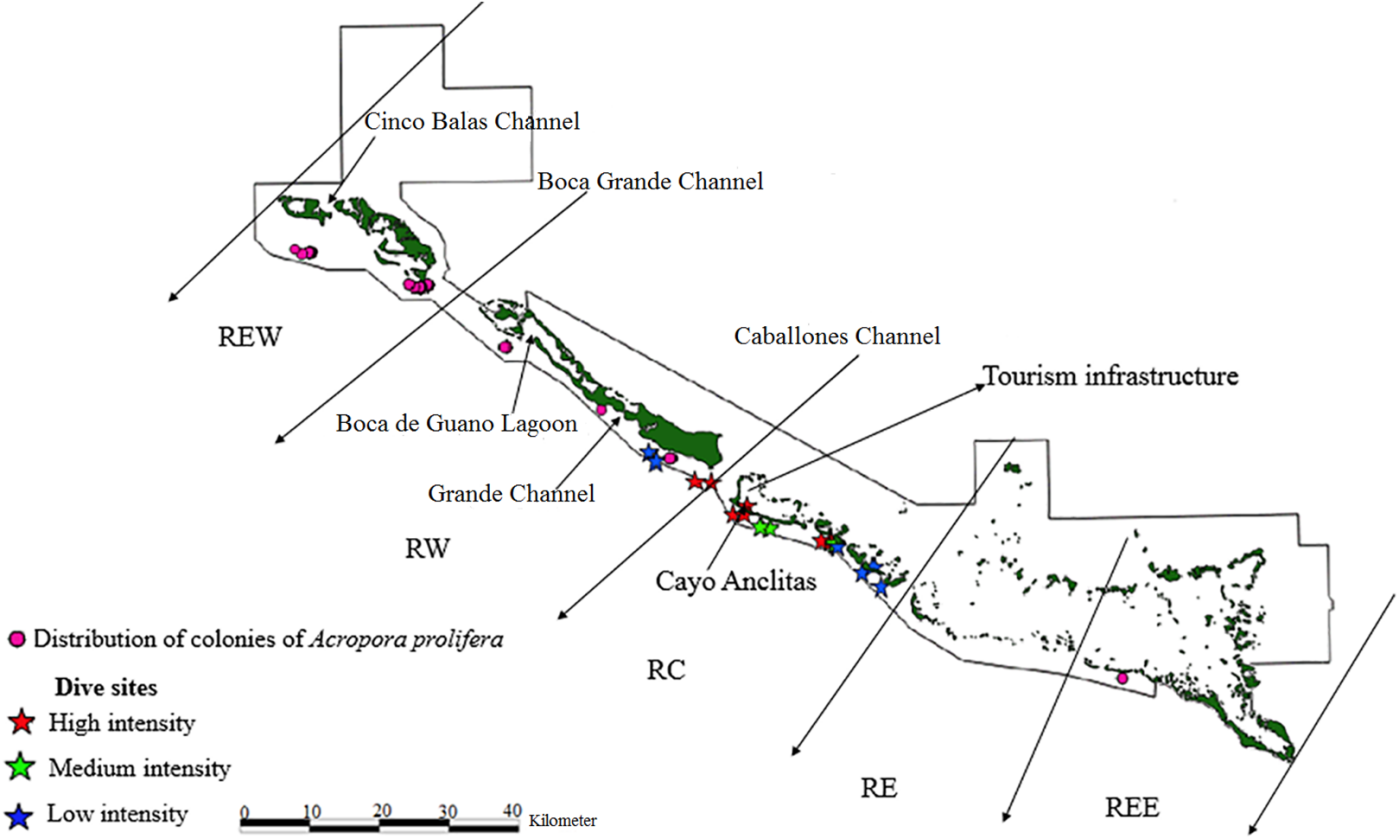
Distribution of live colonies of *Acropora prolifera* in Jardines de la Reina National Park, Cuba.

The limits of the JRNP were taken into account for the distribution of fragments. To stratify our survey, we divided the study area into five zones (Figs. 2–4): Reserve Extreme West (REW), Reserve West (RW), Reserve Center (RC), Reserve East (RE) and Reserve Extreme East (REE). Based on Pina-Amargós et al. (2008, 2014), reserve enforcement follows this zone pattern: RC > RW > RE >REW > REE, where RC has high protection, RW and RE moderate protection, and REW and REE are the least protected. In addition, based on a previous study by Hernández-Fernández et al. (2016), we took into account highly used diving sites and classified their use as low, medium and high intensity.

To determine the status of the *Acropora* spp. colonies, an evaluation was carried out using the criteria of Alcolado & Durán (2011), consisting of a system of scales for the classification and recording of the condition of the benthos and ichthyofauna of the coral reefs of Cuba and the Greater Caribbean region. The following criteria was used: percentage of recent mortality (RM) (%) (critical: >16, poor: 8–16, not good: 4–7.9, good: 2–3.9, very good: <2). RM shows coral reef status last year (McField & Kramer, 2008). According to McField & Kramer (2008), a reef’s health is bad, and a “Red Alarm” is recommended when RM is greater than 5%, for which reason we used this criterion to evaluate the health status of *Acropora* colonies.

The status of 3,546 colonies of *A.palmata* (51%), 168 of *A. cervicornis* (67%) and 104 of *A. prolifera* (100%) was evaluated. The percentage of old (OM) and RM and the presence of bleaching (BL), white pox disease (WPD) and white band disease (WBD) were recorded using ID cards (Weil & Hooten, 2008). WPD and WBD were included in RM evaluation. The maximum diameter was also measured in 4,399 live colonies of *A. palmata*. Size ranges were established (between 10 cm and 50 cm, between 51 cm and 100 cm, greater than 100 cm, greater and equal to 200 cm). The maximum diameter was measured taking as a reference the tips of the most distal branches of each colony.

In order to analyze the influence of location and sea currents on the number of *A. palmata* colonies, reef crests were divided into 500 m segments. All recorded colonies were grouped talking into account the coordinates of the midpoint of their segment and treated as a response variable. The predicting variables were extracted from a detailed map using the Geographic Information System software QGIS 3.0.0 (QGIS Development Team, 2018). They included the coordinates of the segment midpoints, the shortest distance from these points to the mainland, to the closest channel eastward and the specific zone of the archipelago. To assess the potential influence of small-scale oceanographic processes, we explored the relationship between the distribution of the colonies and the distance to the western large channels. In Jardines de la Reina, the reef crests receive greater influence from marine currents out of the west due to their east-west circulation pattern in the south- central Cuban shelf (Claro, Lindeman & Parenti, 2001). In addition, the slope of the line defined by the colonies in each segment was measured to evaluate the orientation of the reef crests with regard to the marine currents.

Before applying any statistical model, data were reviewed to determine if a Poisson or negative binomial distribution were the most adequate to count colonies per segment. Then, collinearity between the covariates was evaluated using the analysis of variance inflation factors (VIF) in a generalized linear model with negative binomial distribution. To evaluate collinearity, the VIF> 2 (Graham, 2003) was used. The coordinate axes were highly correlated with each other, so the distance from the east end of the area to each segment was used as a substitute for both axes. Besides, the specific zone of the JRNP and the distance to mainland were also eliminated because they were highly collinear.

Because preliminary exploration indicated possible nonlinear relationships between the response variable and the covariables, generalized additive models (GAM) were applied. The final model used was a zero-truncated GAM (Zuur et al., 2009) with a negative binomial distribution, because it was the most effective one, based on the Akaike Information Criterion (AICc, Burnham & Anderson, 2002). The decision to use the zero-truncated model was made because the response variable only included segments with *A. palmata* colonies (i.e. no segment with zero colonies was analyzed) and the assumption of a negative binomial distribution can be problematic, since it includes zeros within its range of possible values. If the response variable does not contain zeros, the estimated parameters and the standard errors obtained with a generalized model are likely biased (Zuur et al., 2009).

Graphs of model residuals against the predicted values, and latitude and longitude axes indicated that the model was fit. In addition, a Moran’s I correlogram constructed with the residuals showed that the spatial autocorrelation observed in the raw data was adequately modeled. All analyses were carried out using the software R 3.4.3 (R Core Team, 2017), the zero-truncated GAM model was adjusted with the VGAM package (Yee & Wild, 1996; Yee, 2015) and the Moran’s I correlogram with the NFC package (Bjornstad, 2016).

Additionally, we explored the distribution pattern of *A. palmata* using the Besag’s L-function (Besag, 1977), a transformation of Ripley’s K-function, useful for classifying a point pattern as random, clustered, or regular (Baddeley, Rubak & Turner, 2015). The inhomogeneous L-function was applied after testing the inhomogeneity assumption with the studentized permutation test of Hahn (2012) over 9,999 permutations (Tbar = 1001.5, *p* = 0.6421). To test for significant deviations from a complete spatial randomness, we computed global confident intervals using the Loh’s bootstrap (Loh, 2008; Baddeley, Rubak & Turner, 2015), over nine simulations. The analyses were made in R using the spatstat package (Baddeley, Rubak & Turner, 2015).

## Results

Surveys were performed along some 55 kilometers; approximately the linear distance of the reef crests of the JRNP, out of a total of about 120 km, and roughly the distance from Cabeza del Este to Cayo Bretón. About two km were considered promontories (groups of colonies that build structure, but do not form crests) with *Acropora* spp. In the East sector of the JRNP, the reef crests stretched close to the Piedra Chiquita Channel (Fig. 1). From this point on, we observed isolated *Porites* spp. and abundant standing dead colonies of *A. palmata*.

Towards the West sector, the reef crests showed a much more consolidated formation than in the East sector and were not separated. For this reason, the largest number of colonies, thickets and live fragments of *A. palmata*, *A. cervicornis* and *A. prolifera* were counted in this sector. Nevertheless, abundant standing dead colonies of *A. palmata* were seen. The West sector comprises RE, REW and only four diving sites (two with high and two with low diving intensity) (Figs. 2–4).

There were 7,276 live colonies of *Acropora* spp., of which 104 (1.4%) were *A. prolifera*, 252 *A. cervicornis* (3.5%) and 6,920 of *A. palmata* (95.1%) (Figs. 2–4). There were 104 thickets of *A. palmata*, formed by 8–12 colonies, 3,495 fragments, 22 of which were *A. cervicornis* and the rest *A. palmata* (99.4%). In the East sector of the JRNP, only 263 colonies of *A. palmate* (3.8%), 6 thickets (5.8%) and 32 fragments of *A. palmata* (1%) were recorded. In the same sector, only two colonies of *A. prolifera* (2%) and 11 fragments of *A. cervicornis* (50%) were found.

**Figure 5:**
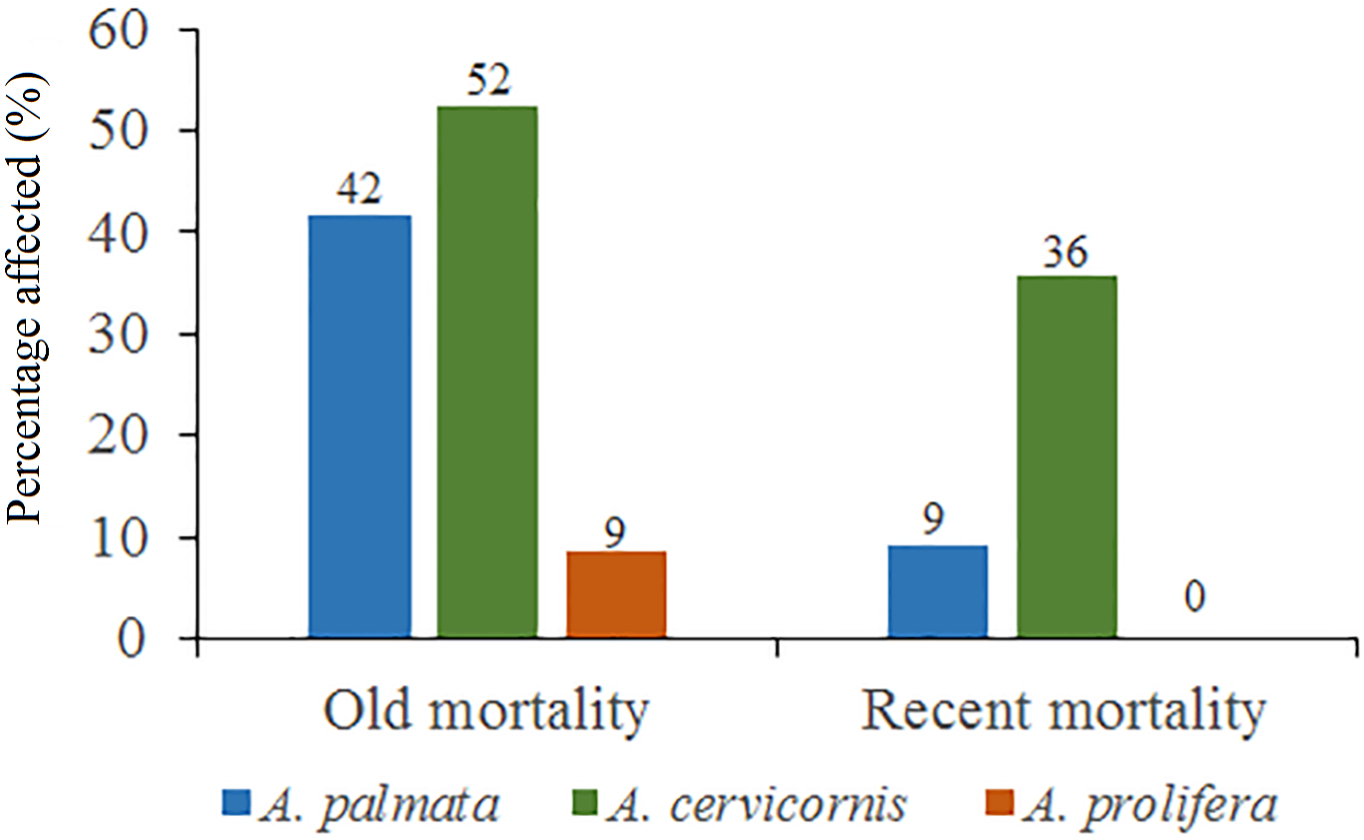
Percentage of old mortality and recent mortality in colonies of *Acropora palmata*, *Acropora cervicornis* and *Acropora prolifera* in Jardines de la Reina National Park, Cuba.

Regarding *A. cervicornis,* 26.2% was affected by BL and 0.6% by WBD. This species showed a high percentage (52%) of OM (Fig. 5) and was only at RW and REW. The highest OM was observed in RW (46.5%), while in REW, it was 7.4%. RM affected 34.6% of the colonies in RW (24.4% BL and 0.6% WBD). The status of *A. cervicornis* was critical, as 30.2% of the colonies were affected by RM (over 16%). In RW, alarm bells should be rung for this species.

Of the 104 colonies of *A. prolifera*, only 9% were affected by OM (Fig. 5) and no diseases nor RM were detected, suggesting that *A. prolifera* is in very good health. Only 2% of *A. prolifera* colonies were affected by BL.

**Figure 6:**
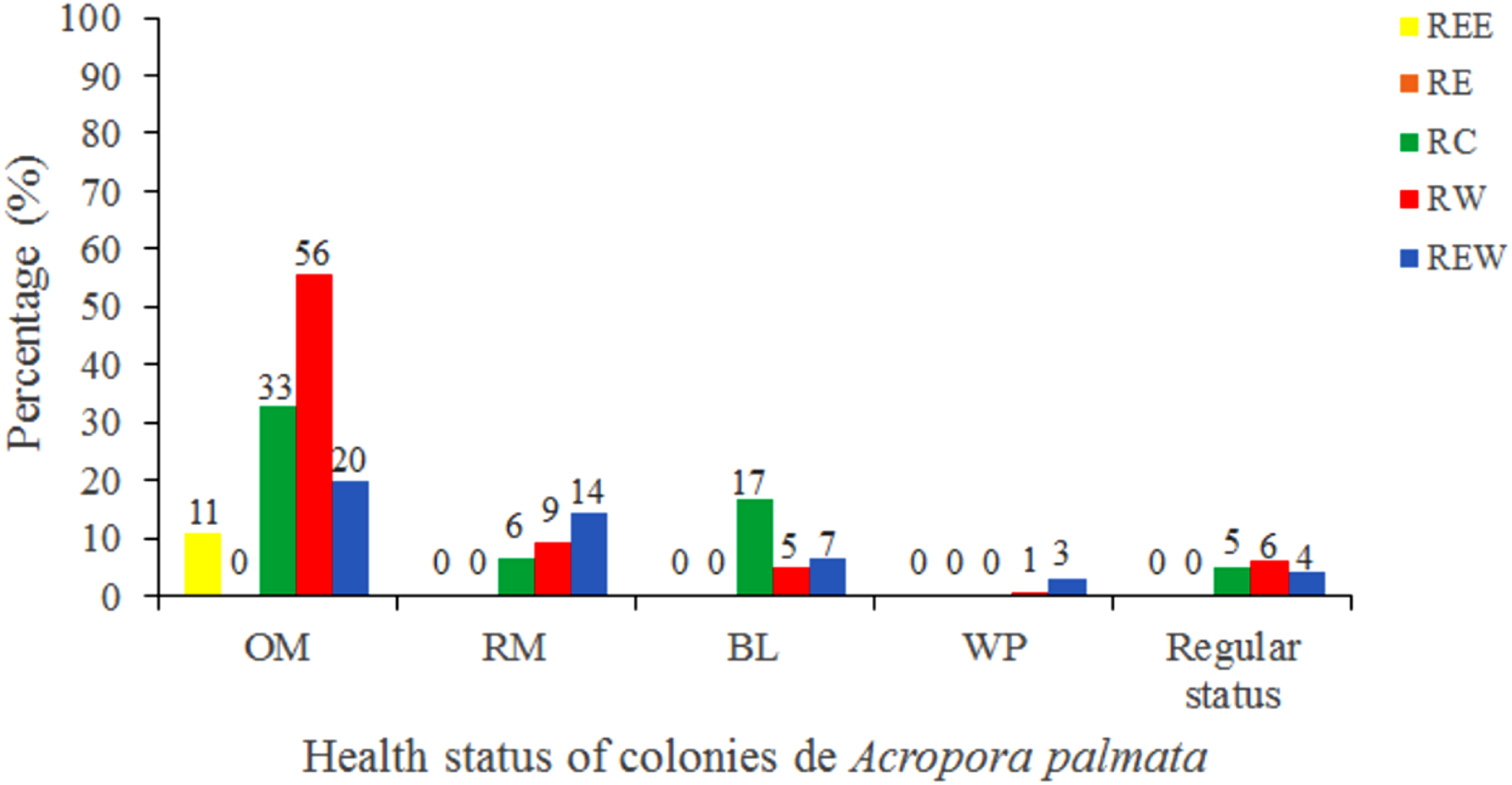
Health status of colonies of *Acropora palmata*, in five reserve zones, in Jardines de la Reina National Park, Cuba. OM: Old Mortality; RM: Recent Mortality; BL: Bleaching; WPD: White Pox Disease.

**Figure 7:**
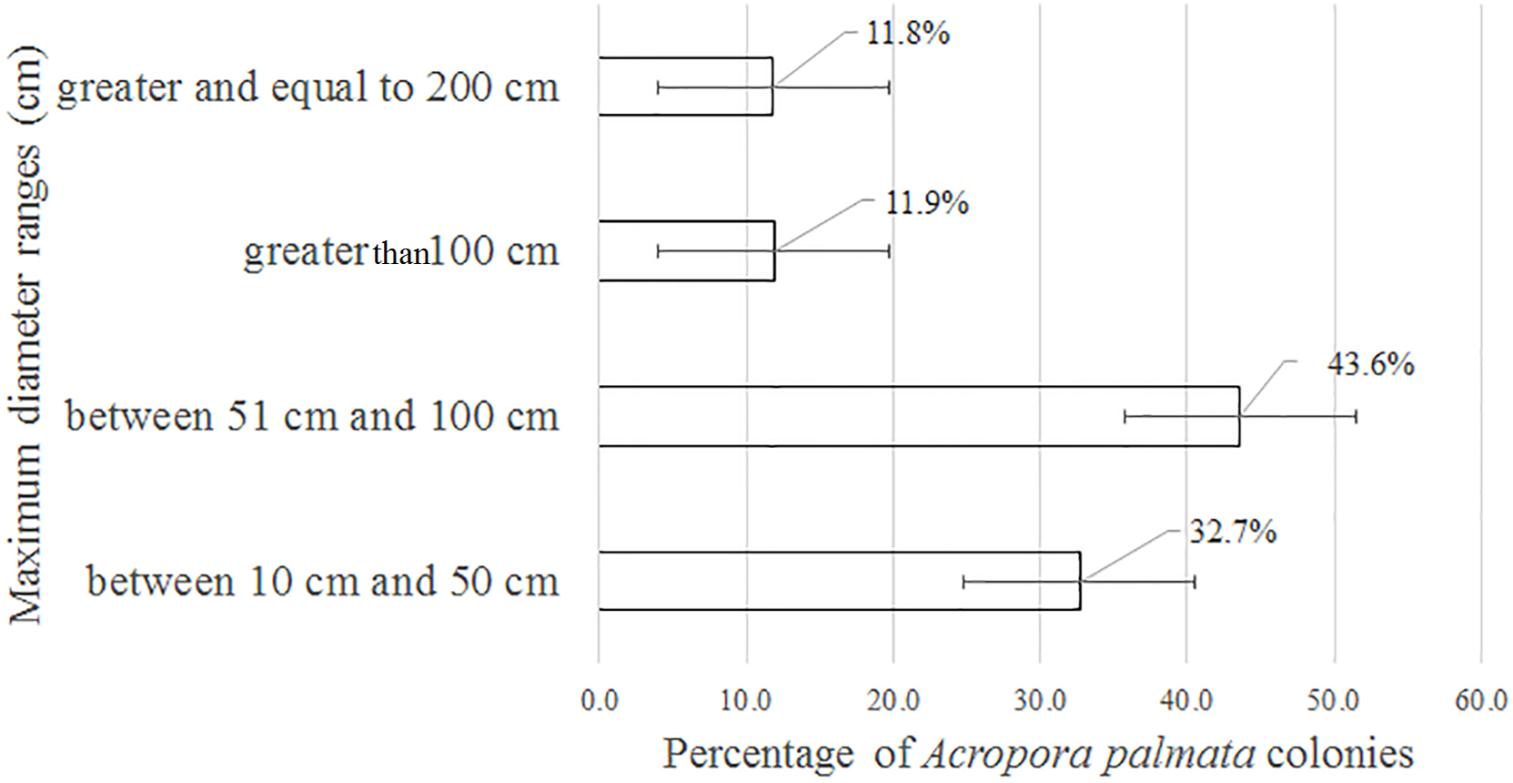
Maximum diameter ranges in *Acropora palmata* colonies in Jardines de la Reina National Park, Cuba.

Of the 3,546 *A. palmata* colonies evaluated, 6% was affected by BL, 1.3% by WPD and 0.3% by WBD. The OM was high in *A. palmata* colonies (Fig. 5), with greater mortality (56%) in RW, followed by RC (33%). Colonies in REW were more affected by RM and WPD. The negative effect of BL was greater in RC (Fig. 6). The health status of *A. palmata* was not good in RC, RW and REW (between 4% and 7.9%) zones and good in RE and REE. This species could be in “Red Alarm” in the RC and RW zones. The maximum diameter of the majority of *A. palmata* colonies (63.5% measured) ranged from 0 to 100 cm (Fig. 7).

**Figure 8:**
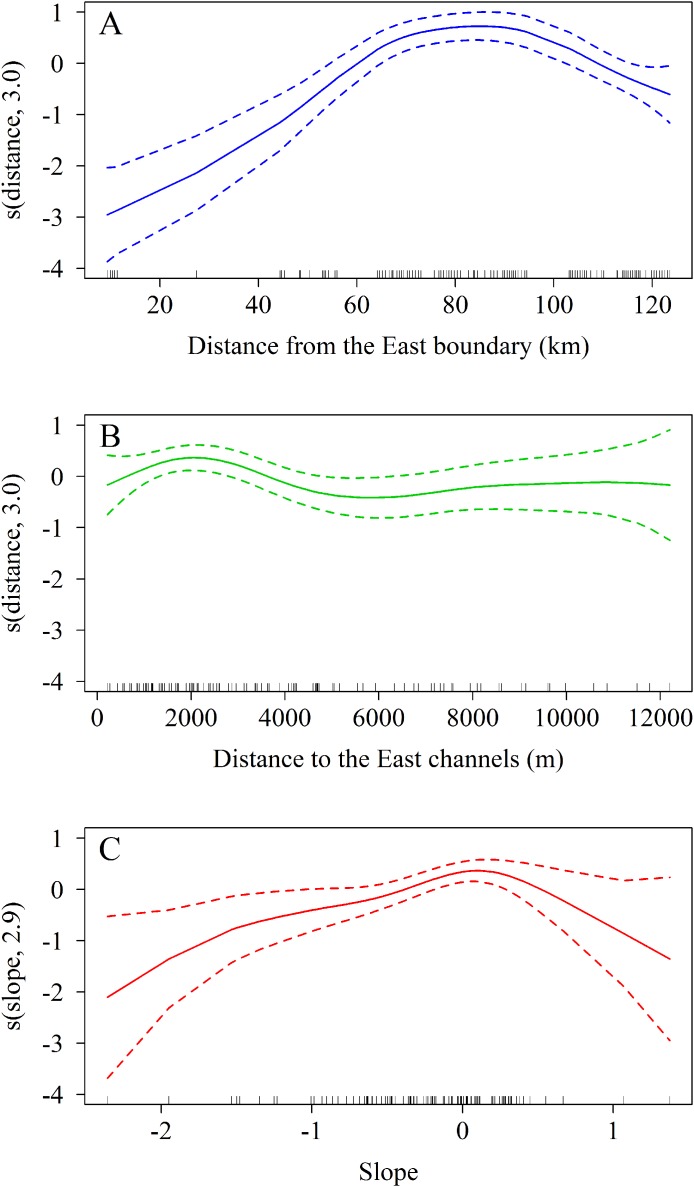
Results of truncated zero GAM applied to *Acropora palmata* colonies respective to geographical position. (A) The distance from the Eastern limit of the study area. (B) The distance to the channel closest to the East of the reef crests. (C) The slope (orientation) of the reef crests. The data was analyzed with a zero-truncated generalized additive model (GAM) with a negative binomial distribution. The solid line indicates the smoothed trend and the dashed lines ± 2 the standard error.

The zero-truncated GAM with negative binomial distribution showed that the number of *A. palmata* colonies varied significantly with regard to changes in the three predicting variables evaluated. Starting from the eastern end of the sampling area, a marked increase in the number of colonies was observed westward (Χ2 = 11.5, df = 3.0, *p* = 0.009, Fig. 8A), which corroborates the existence of a significant difference between the East and West sectors of the archipelago. In addition, there is a negative relationship between the number of colonies and the distance to the channels (Χ2 = 65.0, df = 3.0, *p* < 0.001, Fig. 8B). The influence of the channels is greater within the first 2,000 m (from east to west), where colonies are more abundant; abundance decreases up to approximately 6,000 m, followed by a non-significant increase beyond the latter distance. Finally, the orientation of the reef crests significantly influenced abundance (Χ2 = 15.5, df = 2.9, *p* = 0.001, Fig. 8C). When the reef crests have a horizontal position in regard to the coordinate axes (zero slope), the number of colonies increases significantly when compared to reef crests rather vertical to the axes.

**Figure 9:**
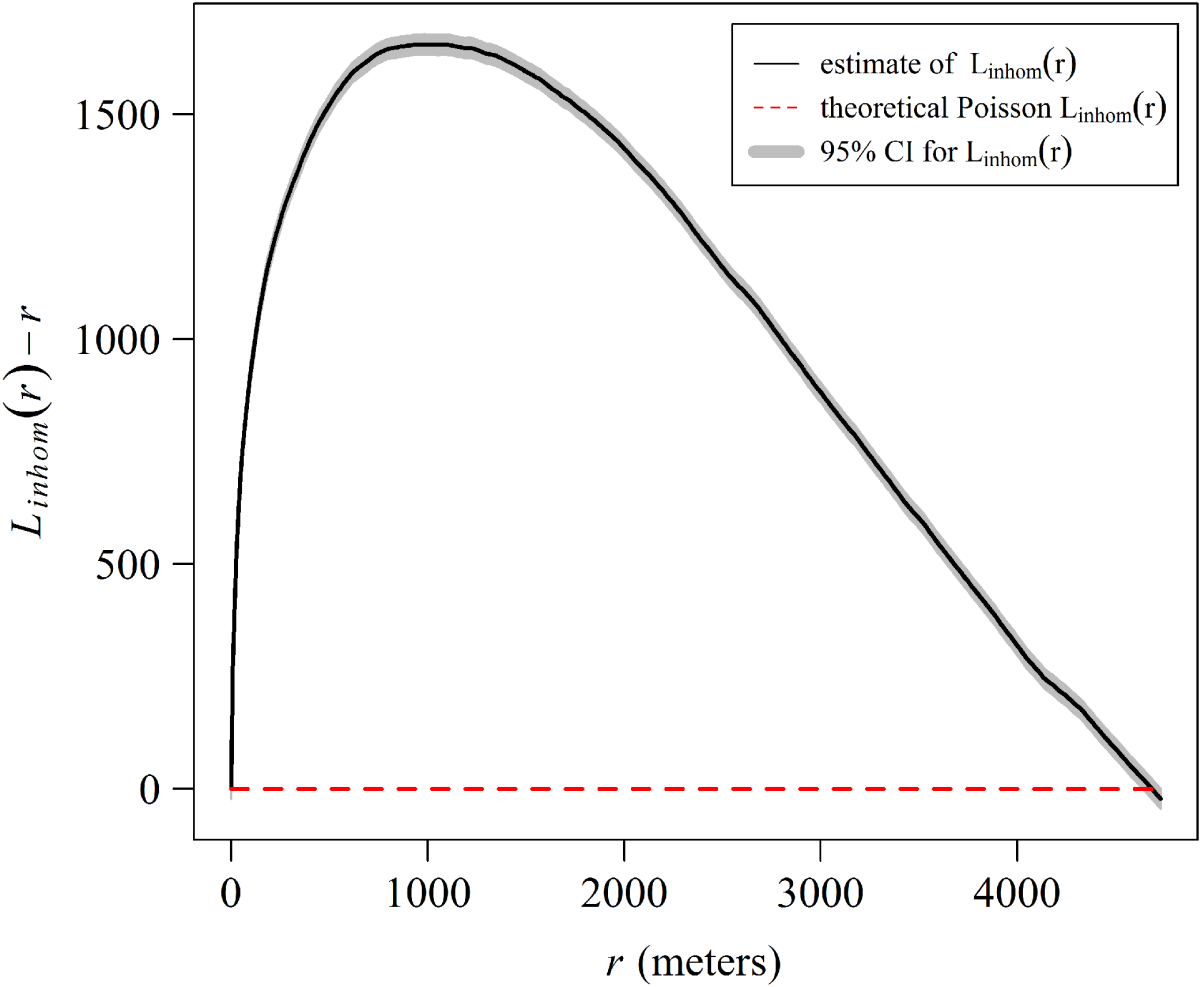
Estimate of the centered inhomogeneous L-function (solid line) for the distribution patterns of *Acropora palmata* colonies in Jardines de la Reina National Park, Cuba.

The spatial analysis allows us to graphically examine the distribution patterns of *A. palmata* colonies. The aggregated pattern over a scale of 4,000 m, has a stronger tendency in the first 1,000 m (Fig. 9). The shaded area in the graph represents a 95% confidence interval for the estimated function, using Loh’s bootstrap (Nsim = 9,999), and the dashed red line is the theoretical inhomogeneous L-function for a Poisson Process (completely spatially random pattern). The higher curve for the estimated inhomogeneous L-function in respect to the theoretical function indicates a significant aggregated pattern in the distribution of *A. palmata* colonies.

## Discussion

The methodology used to understand the distribution and condition of the live colonies of *Acropora* spp. in this study, opens a new approach to marine cartography, allowing for greater precision while assessing changes in the populations (recovery or deterioration) over time (Devine, Loomis & Rogers, 2002). According to Miller, Kerr & Williams (2016), this methodology more efficiently shows the distribution of colonies and live thickets of *Acropora* spp. In this case, the fragments were also taken into account, because they play an important role in the maintenance of local populations and the formation of new colonies (Jackson, 1977). Our study lays the foundations to follow-up the living fragments recorded and their regeneration capacity in the JRNP. As stated by Martínez & Rodríguez-Quintal (2012), the presence of fragments suggests that asexual reproduction may be the principal mechanism of *A. palmata* to maintain and expand its population in the JRNP, allowing the new colonies to be distributed in thickets around the living parent colonies. However, Roth, Muller & van Woesik (2013) stated that the coral fragmentation may indicate the presence of unfavorable environments, since high fragmentation rates give the false impression of expanding and diversifying populations, when populations may be simply cloning.

The decline in coral abundance in the Caribbean region is greatly due to the dramatic loss of *Acropora*. Acroporid populations have declined 80–90% throughout the Caribbean and the Western Atlantic since the late 1980s (Bruckner, 2002). Decline of *Acropora* populations also occurred in Cuban coral reefs between 1987 and 1992 (Claro, 2007). Contrary to the *Acropora* decline in the Great Caribbean (due to WBD), Bruckner (2002) and Claro (2007) found that in the southern coast of Cuba,* Acropora* populations showed low evidence of mortality due to WBD (Rey-Villiers et al., 2016).

More colonies, thickets and live fragments of *Acropora* spp. have been counted towards the West sector of the JRNP. This could be due to the topographic differences of the archipelago in both sectors. Probably, the east-westward orientation of the archipelago, according to González de Zayas et al. (2006), can be an indication of the movement and deposition of sediments and even of the age of the reef crests, the easternmost ones being the oldest. Another observation that could explain the spatial distribution of *A. palmata* is that the easternmost part of the JRNP is closest to the mainland (around 30 km) and the Gulf of Guacanayabo, with higher nutrient content than the Gulf of Ana Maria. Lluis Riera (1977) and Betanzos-Vega et al. (2012), suggest greater inputs rich in organic matter, nutrients and sediments from the mainland in the first gulf. The West sector of the archipelago is more than twice farther from the mainland than the East sector. According to Arriaza et al. (2008), the maximum speed of the currents in the ebb tide (26 cm/s) and the flood tide (13 cm/s), as calculated by hydrodynamic modeling on the SE Cuban platform, were located in the periphery of the confluence of the Gulf of Guacanayabo with the Gulf of Ana Maria. This suggests that the reef crests of the East sector of the JRNP may be subject to greater physical impacts from the sea, likely to increase with extreme weather events.

Although live *A. palmata* was documented in the reef crests in the entire park area, in the West sector there were abundant colonies with 100% OM, especially those far from the tidal exchange channels, where the cays block the process. These standing dead colonies suggest the importance of old populations as habitat for other reef organisms (Martínez & Rodríguez-Quintal, 2012).

**Figure 10:**
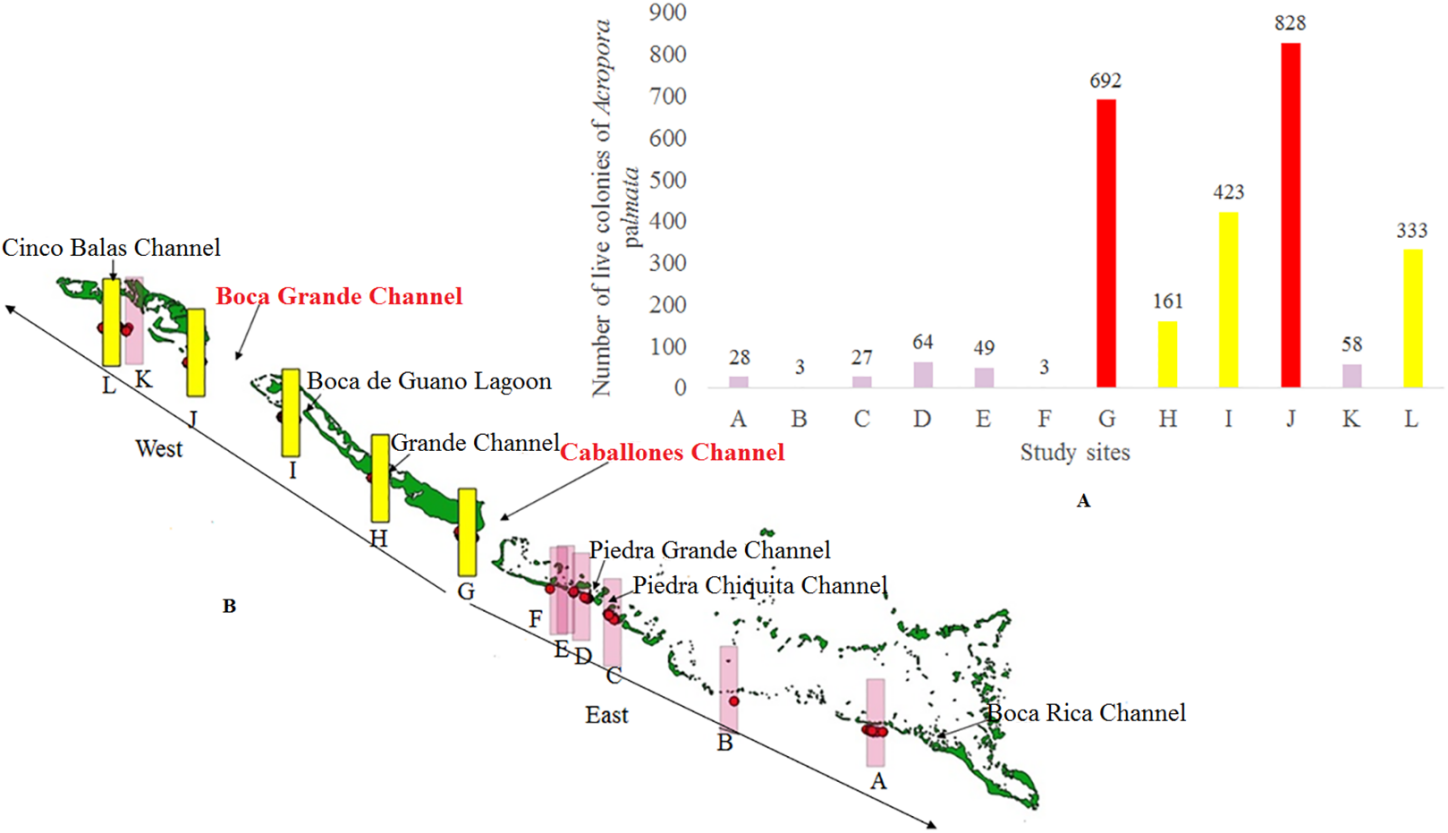
Number of colonies of *Acropora palmata* at 2,000 m from the channel located to the east in Jardines de la Reina National Park, Cuba. (A) In a graphic. (B) Location of all colonies.

An alternative explanation for the different distribution of *Acropora* spp. between the West and East sectors might be that the reef crests of the JRNP act as a barrier, a hypothesis already stated by González-Ferrer (2004). In fact, the pattern described through the GAM model (Fig. 8B) showed that the largest number of colonies (located between the chosen channels) were concentrated 2,000 m away from the eastern channels, where the ebb tide currents from the Gulf of Ana Maria may arrive with greater strength. Due to the Coriolis Effect, the ebb tide currents tend to deviate to the right (west of the cays) and this influence may keep an ideal balance for reef stability in terms of nutrient content, light and organic matter. This behavior is present even further in the channels with greater exchange such as Caballones (approximately three km wide) and Boca Grande (approximately eight km wide) (Fig. 10). The aggregated pattern suggested by the spatial analysis is consistent with the clustered distribution observed in the first 1,000 m from the east side of the channels. This corroborates its influence over the distribution of *A. palmata*, enhancing the density of colonies near these channels.

According to Iturralde-Vinent (pers. comm. Manuel Antonio Iturralde Vinent. 2017), the issue of whether the reef crests or the keys of the PNJR formed first is not resolved. The cays are regarded as an accumulation of sand bars that eventually united. Sand was transported by currents, swell or wind from the lagoon or seagrass beds located between the cays and the reef crests. Based on this concept, the reefs must have formed almost simultaneously with or just before the cays began to form in the Upper Pleistocene to the Holocene. In the Caribbean, the first reefs were formed during the Oligocene, reaching a development peak during the Miocene (field observation of Iturralde-Vinent in González-Ferrer, 2004). However, the first record of *Acropora* spp. as a dominant reef structure dates back to the Late Oligocene (Wallace & Rosen, 2006).

There was no evidence that reserve zones influence *Acropora* spp. populations. Diving sites with higher activity and tourism infrastructure are in RC (where protection is more effective). However, *A. palmata* (the only species present in all reserve zones) has a larger number of live colonies in RW and REW (West sector), where protection levels are lower. There were *A. cervicornis* populations only in RW (in critical status) and REW. *A. prolifera* populations were also found in higher numbers in these regions, and were healthier than any other species. According to Hernández-Fernández et al. (2016) local SCUBA diving does not affect *Acropora* spp. populations, as it is performed 8–22 m deep (far from shallow *Acropora* spp. populations).

The differences in the distribution of live colonies of *Acropora* spp. could be the result of propagation of larvae from *A. palmata* populations located further east than those zones where *Acropora* spp. is scarce (RC, RE and REE). Marine currents mainly flow from East to West and can limit the arrival of new larvae. It is also likely that substratum differences are the cause of different recruitment rates and/or post-settlement different mortality across the sites Zubillaga et al. (2008).

According to McField & Kramer (2008) and based on the RM health indicator, *A. palmata* populations would have been in a “Red Alarm” state in RC and RW, while according to Alcolado & Durán (2011) their health would have been regarded as normal. However, the status of *A. cervicornis* was critical and in “Red Alarm” as well. Regarding health status of the three species, *A. cervicornis* was the worst and *A. prolifera* (with few colonies in the PNJR) was the best.

Fogarty (2012) stated that in some Caribbean sites, *A. prolifera* was found in densities equivalent to or higher than those of at least one were of the parental species. In the JRNP, *A. prolifera* only represented 1.4% of all colonies, something similar to that of *A. cervicornis* (3.5%). A decrease in the parental species, together with changes in the environment, can affect the frequency of hybridization (Fogarty, 2012), which demands further protection and conservation efforts in the case of *A. cervicornis*.

The WBD has been strongly related to thermal stresses resulting from climate change and seemed to proliferate on *Acropora* spp. (Randall & van Woesik, 2015). WPD has been suggested as the principal cause of mass mortality of *A. palmata* within the FKNMS (Patterson et al., 2002). In our study, the impacts of WBD and WPD in colonies of *A. palmata* were low, 0.3% and 1.3% respectively, similar to those reported by Larson et al. (2014) for the reefs of Veracruz (Gulf of Mexico). WBD disease impact was low in the PNJR when compared with results found by Zubillaga et al. (2008) at Los Roques National Park (between 0.39% and 4.69%). However, RM was high (9%) when compared to that obtained by Schelten et al. (2006) (1.33%) for the populations of the southern coast of the Turks and Caicos Islands. RM was higher than that reported by Rey-Villiers et al. (2016) for all coral species in the crests of the PNJR in 2001 and 2012 (≥2%).

In their study of the reefs of Cuba, Alcolado et al. (2003) stated that the species *A. palmata* showed high mortality along the northern and southern coasts of the island. Rey-Villiers et al. (2016) compared some results from the CUBAGRA Project with their 2012 results, and found that OM (for all coral species) was higher in 2012 than in 2001, with prevalence of young corals. Rey-Villiers et al. (2016) stated that in 2001coral cover was low in reef crests, using as a reference the high mortality of *A. palmata* populations. Nevertheless, the authors attributed certain recovery of the species to “over-sheeting”. Bruckner (2002) suggested some evidence of recovery (e.g., southern coast of Cuba), where stable populations were found. Claro (2007) explained that instead of growing and branching independently, in Cuba, new corals of this species were growing on the large skeletons of dead corals, which favored faster recovery. Taking into account references, previous studies and our results, we can infer that a certain recovery of *A. palmata* populations has occurred in the PNJR.

According to Jaap (2002), within the morphometric measurements of *A. palmata*, a very large colony is considered one that reaches 400 cm in diameter among the tips of the most distal branches. In this study, colonies larger than 500 cm were counted, but none reached the maximum diameter of 1,000 cm, as reported for the Montecristi Barrier Reef National Park in the Dominican Republic (Geraldes, 2002). Colonies from 51 to 100 cm were predominant in the JRNP. Taking into account the scale suggested by Rogers et al. (2002) to establish the size of *A. palmata* (small = 0–25 cm, medium = 26–100 cm, large = >100 cm), the colonies that prevailed in the JRNP can be classified as medium-sized. This can be considered additional evidence that *A. palmata* populations had been recovering from possible impacts experienced during the 1980s; similar behavior detected by Zubillaga, Bastidas & Croquer (2005) in *A. palmata* at Los Roques National Park.

Assuming that the *A. palmata* colonies of the JRNP have a similar growth rate than that estimated by Jaap (2002) for the Florida reefs (between 4 cm and 11 cm per year), and by Quevedo (2002) in Puerto Rico (from 5 to 10 cm per year), the recovery of this species dates back to approximately 10–25 years. According to Rey-Villiers et al. (2016), and to the research experience of the authors in Jardines de la Reina, the recovery of *A. palmata* started 10–16 years ago.

The recovery period of *A. palmata* can also be corroborated by the thesis presented by Baisre (2006) on the drastic reduction of nitrogen contribution to Cuban coastal waters that took place during the early 1990s and suggests the oligotrophication of these waters. The reports of nutrient loads in the region, which began in the 1960s, contained typical levels of oligotrophic waters (0.11–0.20 μM of Soluble Reactive Phosphorus, 0.20 μM of Dissolved Inorganic Nitrogen and 4.6 μM of Soluble Reactive Silicate) and may have increased in the 1980s due to greater use of fertilizers in Cuba, although there is no evidence of the possible increase of such nutrients. After the year 2000, nutrient levels in the waters of the JRNP have only been assessed in specific sites and not in the entire park area. In 2013, stations located at the Caballones Channel showed Soluble Reactive Phosphorus levels of 0.28 μM, 3.3 μM Dissolved Inorganic Nitrogen and 4.7 μM Soluble Reactive Silicate.

The apparent recovery of *A. palmata* might be the result of the lack of severe anthropogenic impacts (sedimentation, coastal development, sewage, etc.), hurricanes, storms, and emerging coral diseases (white pox and necrosis), recognized as major threats to the populations of the Florida Keys, Venezuela and the US Virgin Islands (Patterson et al., 2002; Bythell, Pantos & Richardson, 2004; Patterson & Ritchie, 2004; Rogers, Sutherland & Porter, 2005; Zubillaga et al., 2008).

## Conclusions

The results presented in this work provide basic data for future research on the status of *Acropora* spp. populations in the JRNP, where recovery of *A. palmata* has been observed. Knowledge of the species status and possible threats to the populations of *Acropora* spp. can inform decision makers and other actors to develop and implement conservation actions in the park. Such efforts should also include *A. cervicornis*.

## Supplemental Information

10.7717/peerj.6470/supp-1 Supplemental Information 1 Raw Original data from sampling.

## Additional Information and Declarations

### Data Availability

The following information was supplied regarding data availability: The raw data is available in the Supplemental File.
